# Detecting anchored fish aggregating devices (AFADs) and estimating use patterns from vessel tracking data in small-scale fisheries

**DOI:** 10.1038/s41598-021-97227-1

**Published:** 2021-09-09

**Authors:** Ahmad Catur Widyatmoko, Britta Denise Hardesty, Chris Wilcox

**Affiliations:** 1 CSIRO‐UTAS Quantitative Marine Sciences PhD Program, Institute for Marine and Antarctic Studies, University of Tasmania, Hobart, TAS Australia; 2 Commonwealth Scientific and Industrial Research Organisation (CSIRO) Oceans and Atmosphere, Hobart, TAS 7000 Australia; 3 Center for Marine Socioecology, University of Tasmania, Hobart, TAS 7000 Australia

**Keywords:** Sustainability, Environmental impact, Conservation biology

## Abstract

Monitoring the use of anchored fish aggregating devices (AFADs) is essential for effective fisheries management. However, detecting the use of these devices is a significant challenge for fisheries management in Indonesia. These devices are continually deployed at large scales, due to large numbers of users and high failure rates, increasing the difficulty of monitoring AFADs. To address this challenge, tracking devices were attached to 34 handline fishing vessels in Indonesia over a month period each. Given there are an estimated 10,000–50,000 unlicensed AFADs in operation, Indonesian fishing grounds provided an ideal case study location to evaluate whether we could apply spatial modeling approaches to detect AFAD usage and fish catch success. We performed a spatial cluster analysis on tracking data to identify fishing grounds and determine whether AFADs were in use. Interviews with fishers were undertaken to validate these findings. We detected 139 possible AFADs, of which 72 were positively classified as AFADs. Our approach enabled us to estimate AFAD use and sharing by vessels, predict catches, and infer AFAD lifetimes. Key implications from our study include the potential to estimate AFAD densities and deployment rates, and thus compliance with Indonesia regulations, based on vessel tracking data.

## Introduction

Since their widespread introduction in the early 1980s, artisanal fisheries have relied on Anchored Fish Aggregative Devices (AFADs) as a fishing aid to increase catch rates^1,2^. Various types of AFADs are used in the Indian and Pacific oceans; with deployments from the coastal zone (< 12 nautical miles from shore) extending far offshore out into the deep sea (to depths up to1500 m)^1,3,4^*.* Nowadays, AFADs are mostly deployed in developing countries where they play a vital role to the food security for coastal communities^5–7^. A key concern with AFADs is that most fish caught around AFADs are smaller than those caught using other gear types or fishing practices^8–10^.

In most countries and regional fisheries management organisations (RFMOs), the management of AFADs to date has focused on preventive actions such as temporal closures, regulations of gear types, and licence regimes^11,12^. These actions rely heavily on the compliance of fishers and do not address illegal deployments^13,14^. Moreover, vandalism and conflict over FADs make fishers reticent to disclose FAD locations^6^.

Typically, fisheries authorities have limited tools to conduct control and surveillance when it comes to AFAD management. Direct monitoring in the ocean has proven to be impractical and expensive^15^. The structure of AFADs, with no tracking device and only a small buoy as a position marker, means AFADs are hard to detect at sea^16,17^. Although it has been suggested to attach satellite buoys to AFADs, this option would be hard to implement in small-scale fisheries^15^. Furthermore, aerial surveys, which can cover large sweeping areas are generally too expensive, particularly in developing countries^18^. Hence, identifying lower-cost solutions to monitor AFAD use is of relevance, particularly in small-scale fisheries.

Vessel tracking technologies, such as vessel monitoring system (VMS) transponders, are commonly used to monitor fishing activity and compliance around the world^19,20^ and have been suggested as an approach to infer FAD use^21^. The data obtained from VMS has been used to understand illegal unreported and unregulated (IUU) activities in fisheries management such as transhipment and unauthorised fishing^20,22,23^. However, VMS is currently utilised solely on larger vessels (e.g. > 30 gross tonnes in Indonesia) and such mandatory systems have generally not been required on small fishing vessels to date.

In recent years, low-cost tracking devices have become increasingly available, with devices such as the SPOT Trace ($99.99 USD) allowing satellite-based tracking of individual vessels in real-time for a few hundred dollars per year^24^. This has led to an explosion of their use, as they can be applied to fishing vessel monitoring for small vessels inexpensively, at both the individual and fleet level^21,25^.

The number of AFADs in Indonesian waters remains unknown due to unreported deployments. However, a total of 3858 AFADs has been suggested as the official number of registered AFADs^26,27^. Considering that AFADs have an average lifetime of two year^28^ and they are utilized by fishers employing a variety of gears such as purse seine, pole and line, hand line, trolling lines, and kite line fishing^29^, the actual number of AFAD is likely to be substantially higher. A recent report from Proctor et al., (2019) estimated 5,000 to 10,000 is realistic approximation of total AFADs in Indonesian waters^28^ and another recent estimate suggests there are 10,000 to 50,000 AFADs in operation (Widodo et al., in review)^30^.

The regulation of AFAD deployment in Indonesia was established in 2014^31^, and revised in 2021^32^. Under this new regulation, a single vessel can own up to three different AFADs deployed in Indonesian Fisheries Management Areas (FMAs) and a maximum of 15 AFADs deployed in the high seas. The new regulation also requires fishers to put radar reflector on the AFADs buoy, and every deployment must be reported to the fishing authority. In addition to collective ownership by the small scale fishing community^33^, groups of 10 fishing vessels can have up to five different AFADs. The regulation also requires a minimum distance of ten nautical miles between AFADs. However, many fishers are not aware of the regulation and enforcement from the fishing authority remains limited^34^.

In this paper, we explore the use of inexpensive tracking devices to answer three key questions about AFAD use by hand line small-scale vessels: (1) can we use tracking data on small-scale vessels to estimate AFAD locations and use patterns; (2) can this information be used to predict the relationship between the number of vessels and the number of AFADs, given complexities such as AFAD sharing; and (3) is it possible to infer catches of vessels based on their use of AFADs and other characteristics of their trips?

We address these questions using an analysis of landings and vessel tracking data collected on small-scale handline tuna vessels in three locations in Indonesia (Labuhan Lombok, Oeba Kupang, and Bone fishing ports), in combination with direct observation of a fishing trip by a small-scale vessel. We focus this case study on Indonesia small-scale vessels because Indonesia is a major fishing nation with an estimated minimum of 225,000 small-scale vessels which currently are not monitored because they do not meet mandatory VMS monitoring requirements for vessels over 30 GT^35^. Moreover, AFADs are highly utilized in the small scale fishery which targets pelagic species such as tuna^29^. Hence, understanding small vessel movements and AFAD fishing here has major implications for the neighbouring tuna RFMOs and provides an ideal opportunity to explore this issue that can be applied to understand AFAD use around the world.

## Results

**Figure 1:**
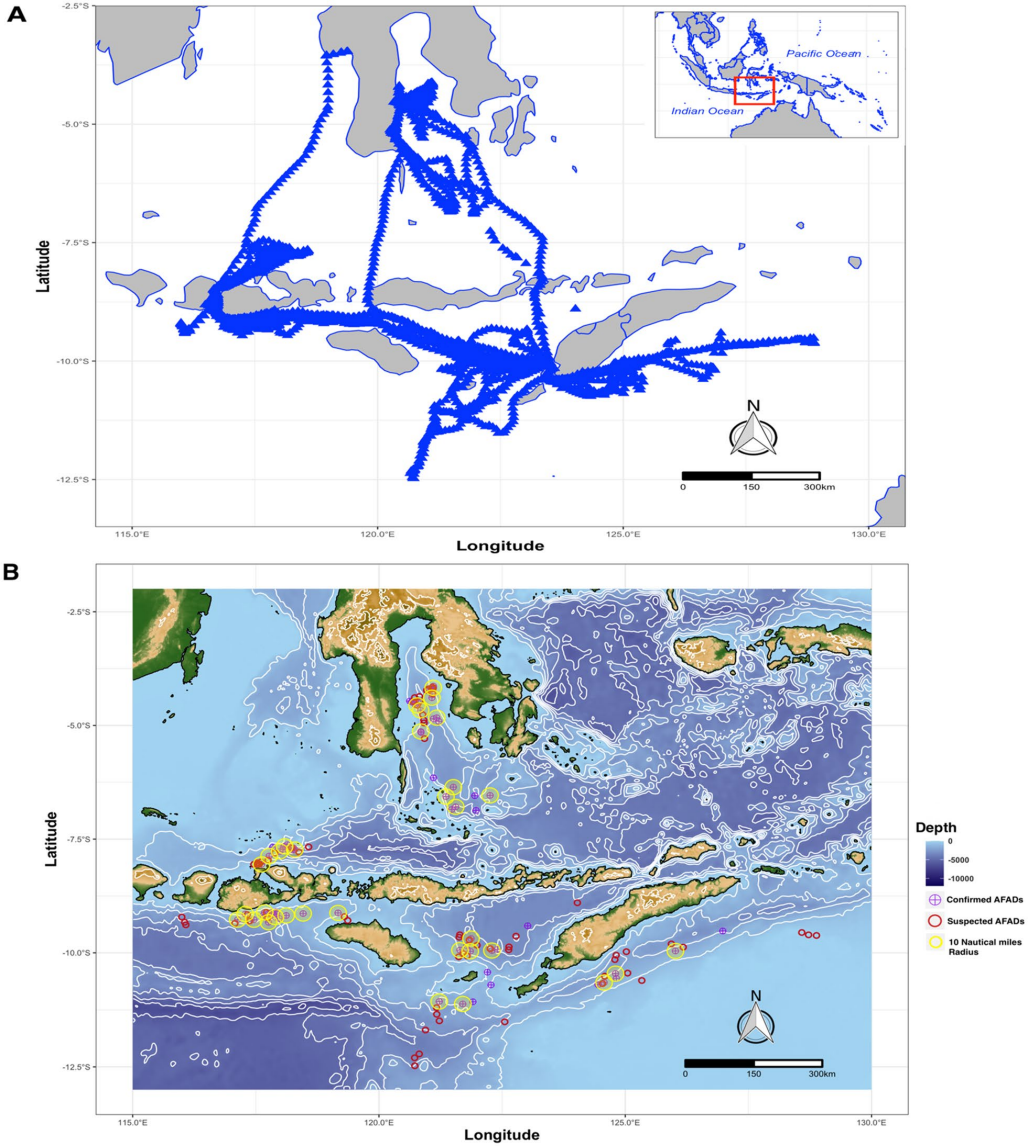
**(A)** Blue triangles indicate raw data of vessels’ tracking records, depicting vessel movement patterns; and **(B)** suspected, and confirmed FAD’s location with ten Nautical miles as indicated from confirmed FADs. Map made using R version 4.0.3^36^ by utilizing marmap^37^ and ggmap package^38^ with bathymetry data extracted from ETOPO1 1 Arc-Minute Global Relief Model^39^.

The initial plot of all vessel tracking records showed that fishers in these three provinces were moving between provinces (Fig. 1A). After segmenting trips and identifying offshore slow speed positions (0.119 km/h on average) from the vessel data, the DB SCAN clustering algorithm identified 139 aggregated areas of positions that represented potential AFADs. The AFAD positions were concentrated into clusters that corresponded with frequent vessel routes (Fig. 1B). Most of the AFADs detected were located less than 10 nautical miles apart (Fig. 1B).

Based on our criteria, a total of 72 confirmed AFADs were recorded (SI, Table 1). We also detected AFADs on the 27 fishing trips which had port sampling records. These were later confirmed by interviews (SI, Table 2). Confirmed AFADs were dominated by deep sea AFADs, typically in depths greater than 1000 m. Overall, only six out of 72 were located in less than 1000 m deep (AFAD depth ranged from 475 m (ID 71) to 3179 m (ID 45)). Furthermore, AFADs were located far from shore, with distances ranging from 10.5 km (ID 15) to 100.2 km (ID 64) (see SI, Table 1).

**Figure 2:**
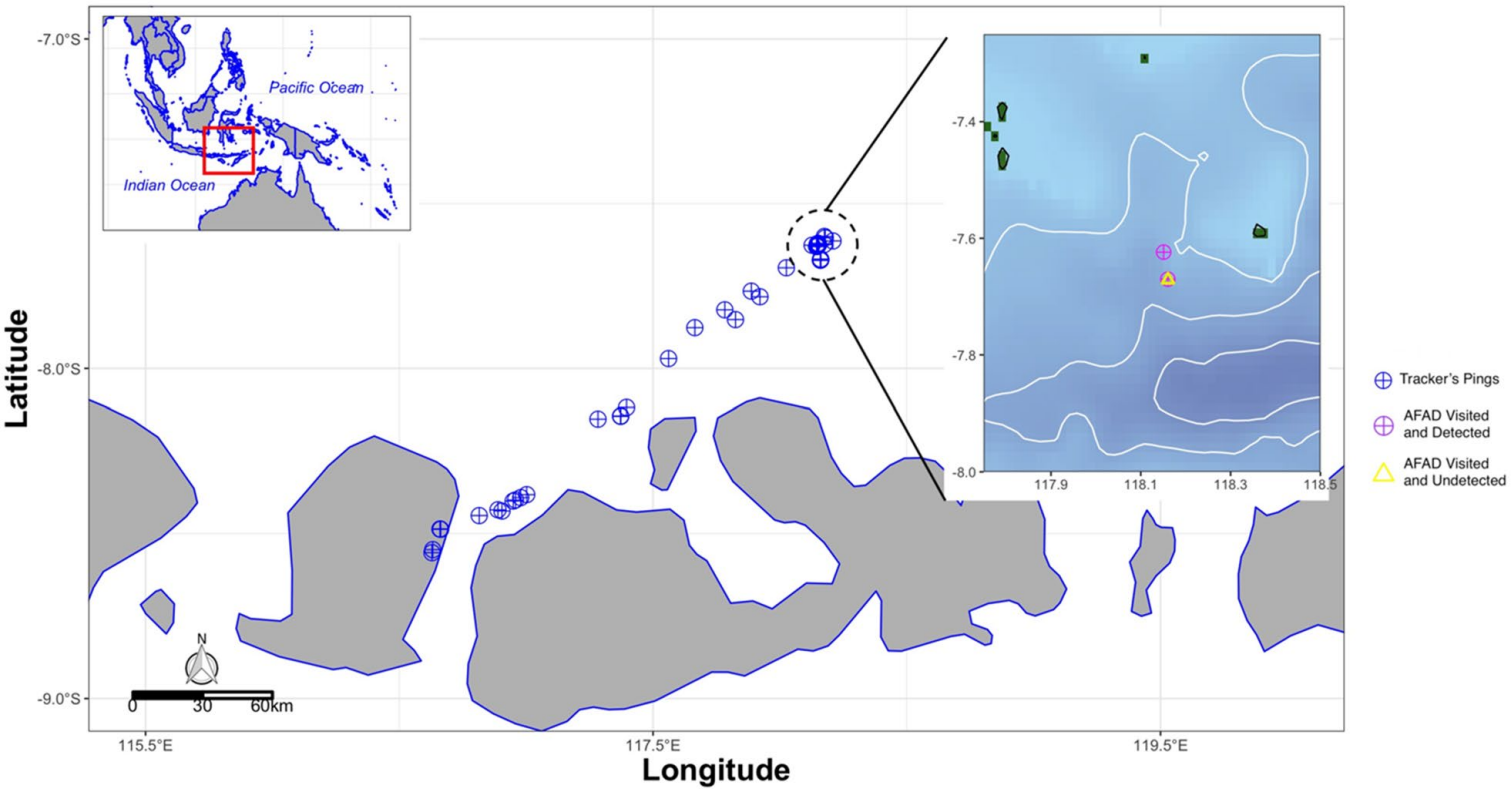
The full record from the SPOT Trace tracking device onboard the vessel, and AFADs visited and analysed by using DBSCAN. Map made using R version 4.0.3^36^ by utilizing marmap^37^ and ggmap package^38^ with bathymetry data extracted from ETOPO1 1 Arc-Minute Global Relief Model^39^.

During the vessel-based ground truth excursion, the vessels visited three different AFADs, two of which were detected by our cluster analysis (Fig. 2). The deepest AFADs recorded on this ground truthing fishing trip was 1408 m, with a distance between first and second AFAD of < 1 km. In contrast, the distance between the second and third AFADs visited was 6.2 km.

**Figure 3:**
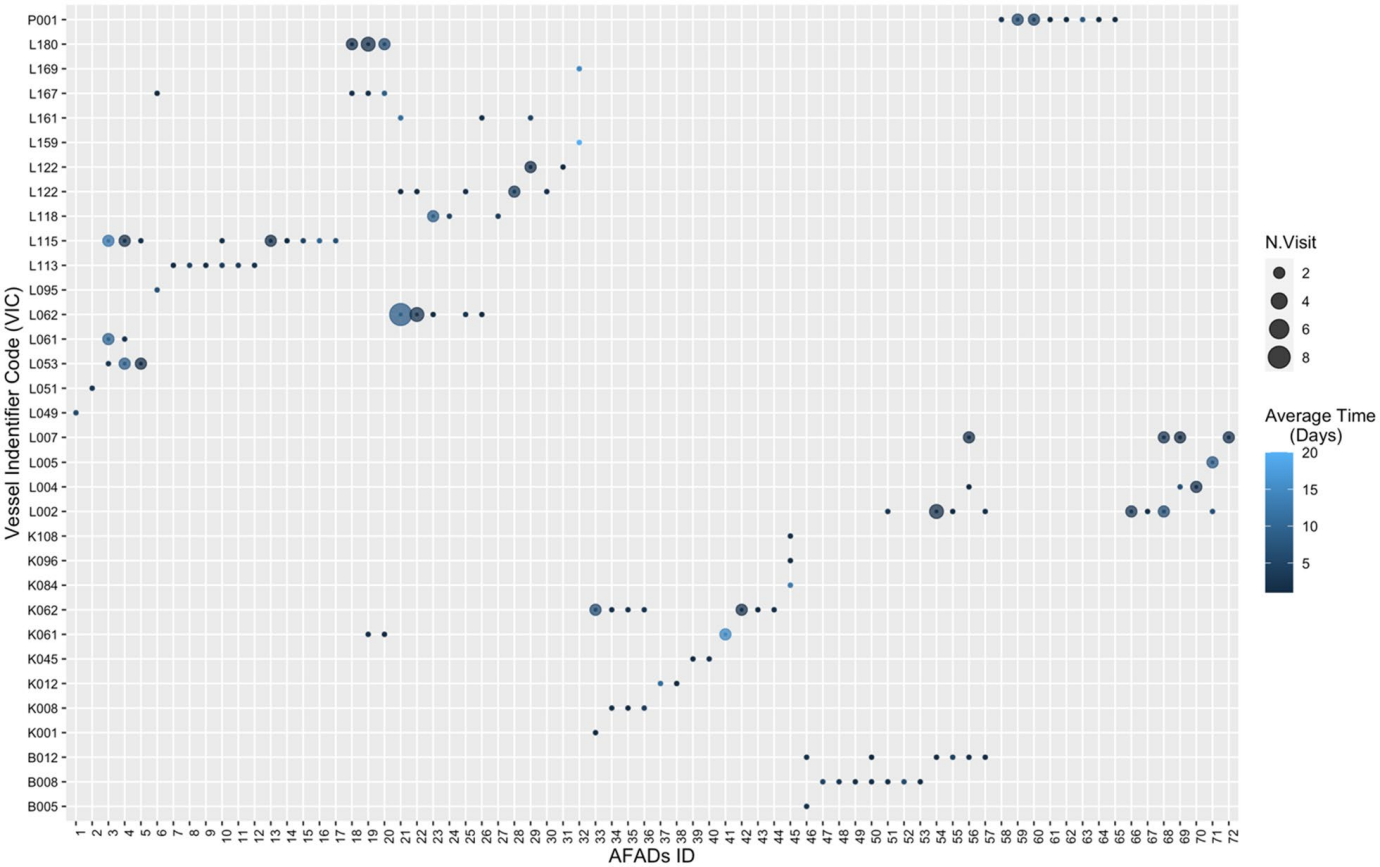
Number of AFADs used by each vessel (shown on the x axis) and the average time each vessel spent fishing on confirmed AFADs during the study period (Days). The number of visits is depicted by the size of the circles, as per figure legend.

Looking at the visitation pattern from the vessel’s perspective, a single vessel (VIC L115) visited up to nine different AFADs during the study period (Fig. 3). A single AFAD was visited by up to 3 different vessels as a maximum. Overall, 32 of the 72 confirmed AFADs also were visited by more than one vessel throughout the study period from our pool of tracked vessels alone (SI, Table 1). We also observed multiple AFAD visits for each fishing trip, with 21 of 27 fishing trips which had port sampling data records (SI, Table 2). However, we also found that a vessel can spend the entire trip fishing only on a single AFAD (see SI, Table 2).

**Figure 4:**
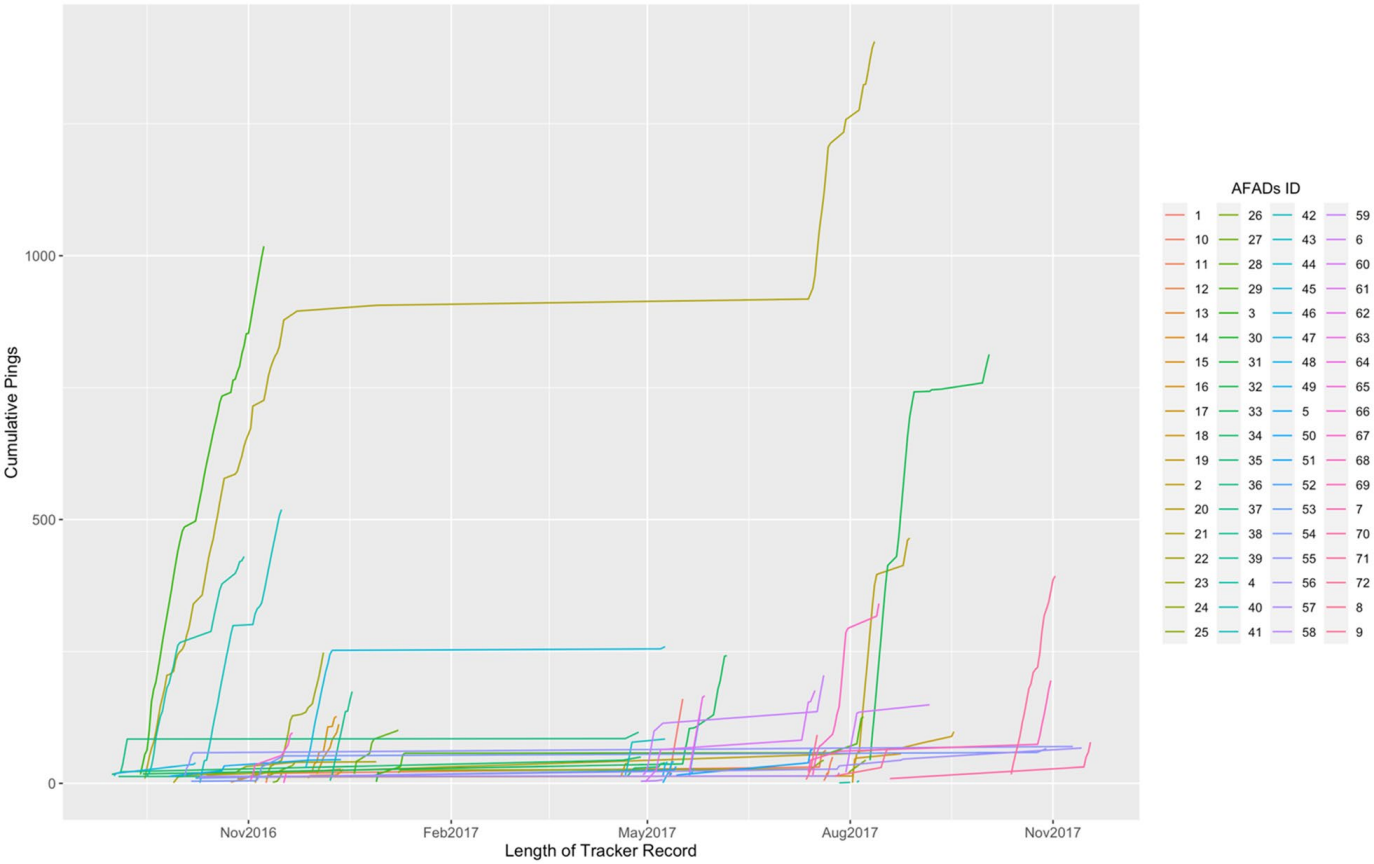
The length of tracker record for each AFAD detected. On the x axis we show the date of the trips, while the y axis shows the cumulative number of ‘pings’ recorded by the Spot Trace device. Colours for the lines indicate different AFADs (as per figure legend).

The visitation periods for individual AFADs in our dataset ranged from one day to up to nearly a year (Fig. 4). Visitation patterns tended to be aggregated in time, with a period of intensive usage, followed by a period of inactivity, and subsequent periods of usage. Most of the AFAD use ended after a period of inactivity on that AFAD, though vessels that had visited a particular AFAD were still carrying tracking devices.

**Table 1:** Relative of models for total catch landed from a fishing trip.

Model	AIC
sFsDrV	373.9
sFrV	387.9
rV	405.4
s(FD)rV	413.3
sDrV	413.9
s(FD)	418.9
Null	420.3

Model codes include *F* number of FADs visited, *D* length of trip in days, *V* vessel identity, *null* intercept only. Functional codes are *s* smooth term, *r* random effect term, parentheses indicate a multidimensional smooth across the included terms.

**Figure 5:**
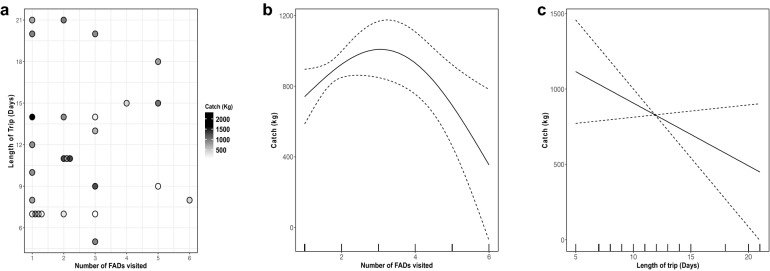
Patterns in catch with the number of FADs visited and the number of days in a fishing trip from the 27 vessels with port sampling data. **(a)** The catch data plotted against the two variables of interest (the number of AFADs visited on the x axis, and the length of the trip in days on the y axis. The points are offset to the right of a value when there are multiple observations, for ease of reading. **(b)** The best fitting model for the data based on AICc for the number of FADs visited and the catch in kg (with a smooth term for the number of FADs visited during a trip). **(c)** The length of the trip in days and the catch in kgs of the best fit model for the data based on AICc.

We found that the best model for the catch landed from a fishing trip included the length of the trip and the number of AFADs visited, along with a random effect for the mean catch by a vessel (Table 1, Fig. 5). Interestingly, fish catch first increased, and then decreased with the number of AFADs visited (Fig. 5b). However, the term was only weakly significant, as illustrated by the relatively wide confidence intervals (Table 2, Fig. 5b). While the term for the length of a trip was not significant, it does suggest that catches also decreased as trips were longer (Fig. 5c). This basic model with two terms explained 95.6% of the deviance in the data and was significantly better than a null model (Null AIC: 420.3, two term model AIC: 373.9). These patterns can be seen in the raw data, despite it not being standardized for the average catch for each vessel. The lower left corner of the plot contains most of the relatively high catches (Fig. 5a).

**Table 2:** Best model for total catch landed from a fishing trip.

Parametric terms			
	Estimate	Standard Error	Pr( >|t|)
Intercept	835.996196	269.760275	0.0175

Smooth terms			
	Effective degrees of freedom	Reference degrees of freedom	Pr( >|t|)
Number of FADs	1.821	1.958	0.097
Number of days	1	1	0.145
Vessel identity	16.241	17	0.004

## Discussion

Technological advancements improve our ability to manage natural resources. This is particularly relevant for small scale fisheries, where there is a need for low-cost data sources to improve our understanding of fishing effort, catch, and the associated sustainability of fish resources required for global food security. GPS trackers have now been widely used to study the behaviour of small-scale fisheries^25,40^. We found focusing on patterns of vessel movement to be a low-cost, reliable approach to identify fishing grounds, as well as to understand both the spatial and temporal usage of AFADs, and ultimately predicting the resulting catch.

We acknowledge that the number of actual AFADs used by our tracked vessels is likely much higher than the number estimated in this study. This is in part due to our requirement for a potential AFAD to have been visited at least two times before we considered it a confirmed AFAD. These criteria significantly reduced the number of AFADs reported (from 139 to 72 AFADs). However, we erred on the cautious side as we were unable to distinguish between AFAD fishing and other non-AFAD fishing behaviours, such as bait fishing, that might involve vessels being stationary. Furthermore, given that the length of trip for a vessel is 5 to 20 days, the one-month period over which a SPOT Trace tracker is deployed means there is a maximum of two fishing trips possible during our observation period. This leads to the potential that even the tracked vessels may have additional AFADs they use outside of the fishing trips observed in this study period.

Another source of underestimation in AFAD numbers may come from the distance parameter we employed in our analysis. During the ground-truthing, only two out of three visited AFADs were detected by DBSCAN. This is because the radius of movement between two of the FADs (Fig. 2) was overlapping. This is possible, as currents and winds displace AFADs synchronously, and thus tangling is reduced, allowing AFADs to be deployed closer together than the sum of their surface radii. Therefore, the distance among vessel positions clustered two AFADs, identifying them as a single AFAD, given the criteria we applied. The implication of this potential for multiple AFADs within a DBSCAN cluster is that the locations we detected could actually represent a much larger number of AFADs that are deployed close together. Future extensions of this work could include estimating the number of AFADs within clusters using the geometric pattern of the boundary of the cluster. For instance, a figure-eight shaped boundary would indicate there are two FADs in a cluster rather than one. However, SPOT Trace deployments would need to be longer to provide adequate data to distinguish this subtlety.

Since our study did not include records from the first time each of the AFADs were deployed, we were unable to determine the absolute lifetime of AFADs in the region. However, based on the vessel tracking data, only a few AFADs were visited for nearly one year implying that AFADs might be failing in less than one year. Because the record of AFAD usage is from the vessel perspective, when the tracker on a vessel is removed at the end of its month long deployment, the record stops while the AFADs may still exist and remain in use. If other vessels in the study use the same AFAD, the record for that AFAD will continue, but if not, it ends with the removal of the tracker from the vessel using it. Hence, the lifespan of AFADs we report is an estimate that should be treated as a minimum lifespan. Moreover, since fishers tend to deploy AFADs in a particular fishing location, it is also possible that the fisher has deployed a new AFAD in the same location. However, given the deployment precision required this may not be as big of a source of error as underestimation.

Conversely, from long periods of inactivity at individual AFADs (as shown in Fig. 4), we suspect that some AFADs may have been lost and replaced over the course of the longer use patterns we observed. These inactivity periods take place during the wet season, which typically has rougher weather and poorer fishing conditions, particularly for small vessels. Hence, we might anticipate fewer vessel days at sea or the loss of AFADs due to failure of their moorings during periods of high swell. The asynchrony in the time at which inactivity patterns begin and end, however, suggests that a lack of fishing activity is unlikely to be the sole source of the observed inactivity periods and that there is likely a contribution from AFAD loss and replacement. With additional tracking data on individual vessels, it might be possible to disentangle these differences by looking for subtle shifts in the centres of the spatial clusters, indicating a new deployment. However, the current observations are inadequate to provide this level of resolution.

The AFAD sharing practices identified in our study reveal a management opportunity to reduce the number of AFADs deployed. The use of AFADs can be maximized by extending the users beyond the owners of an individual AFAD, or by considering AFADs a community resource. While perhaps not suitable in all areas, given that sharing AFAD is relatively widespread, this presents a viable option. Developing a management system that allows limits on the total number of AFADs but provides for a system of rotating access may allow for the establishment of a biologically sustainable system of AFADs whilst minimizing social and economic disruption to the fishers. Moreover, it may also reduce the incentives for fishers to keep AFAD locations private.

The catch data obtained from the port sampling allowed us to identify the factors that influence the total catch. The number of AFADs visited is the main factor that significantly affects the weight of catch by a vessel on a fishing trip, given the average catch of a vessel. Trip success increased as more AFADs were visited, but then declined sharply beyond 3 AFADs. Similarly, for a given vessel, as trip lengths increased, catches were lower.

This pattern might be expected if fishers are considered as central place foragers in the context of the optimal foraging theory^41^. Vessels typically leave and return to the same port. Presumably while at sea, they attempt to either maximize their catch or at least satisfy a minimum required catch to meet their fixed costs. In either event, one would expect fishers to extend their trip length if catch rates are low to try to meet their objective, subject to other constraints such as fuel supply or adverse weather. In this context, if they visit an AFAD and have a low catch rate, one would expect fishers to move to another AFAD. Thus together, the number of AFADs visited and the length of the trip provide a reliable predictor of the quality of a fishing trip, in terms of variation around the average for a given vessel. This information is very useful, as it suggests that the SPOT Tracking data, or other vessel tracking information, can be used as a proxy for port sampling. Thus, remote monitoring of the vessels can be used to get some measure of stock status, via catch rates, or as a check against port sampling or logbooks to check their veracity. Given the rapidly falling cost of technologies, such as the SPOT trackers used in this study, proxies for catch rates such as the one we developed here could facilitate fleet-wide monitoring. In Indonesia, with a quarter-million small scale vessels spread across thousands of islands this scalability is critical, and given Indonesia has the third highest marine catch in the world^42^, the resulting management improvements have global ramifications.

The case of Indonesian FAD management challenges reflects current global FAD management challenges, especially in artisanal coastal fisheries in Pacific island countries where AFADs are commonly used^43^. We found that AFAD deployments in Indonesia are very dense, and frequently well inside the minimum ten nautical miles spacing required by law. Based on our study, it is also clear that vessels are using more than the three AFADs limit allowed in current regulations. These high densities and usage rates could be reducing the effectiveness of AFADs to aggregate the fish by dividing the fish concentration among close AFADs and thus decreasing catch rates. Moreover, the current concentrated use of AFADs could also be leading to large numbers of lost and abandoned AFAD structures, with significant impacts on the ecosystem and local habitats^44,45^. Fishers could deploy fewer AFADs, thus decreasing their potential impacts. The regulation of AFADs in Indonesia, which has been in place since 2014, is still not effectively enforced due to technical issues. Moreover, the users of this type of FAD are dominated by small-scale fishers whose livelihoods and food supplies likely depend on the additional efficiency, making management more problematic.

Expansion of the current study from a monthly sampling approach to continuous monitoring of vessels would greatly improve our ability to discern AFAD use patterns, infer catch dynamics, and ultimately investigate the potential for management strategies that could balance maximizing the benefits from AFAD deployments and controlling their environmental and social impacts. Ultimately minor technological improvements which extend tracking device lifetimes, along with links to other electronic monitoring approaches such as low-cost onboard cameras or electronic logbooks and landing records could allow cost effective monitoring of the vast small scale fleet in Indonesia, leading to better fishery outcomes at a significantly reduced cost. Expanding these approaches, particularly in the case of rapidly falling technology costs, has significant promise for improving management across the many fisheries and sectors in Indonesia, and elsewhere.

Most of the global FADs are managed by the regional fisheries management organizations (RFMOs), and not all member countries have implemented regulations regarding FAD use^46,47^ (IOTC, 2018). Given the large proportion of world tuna production which is dominated by floating object fishing, compared to fishing on free schooling tuna^48^, more investment in FAD management will likely yield an overall improvement in fisheries management and catch sustainability. Paired with addressing management of Indonesia’s very large small scale tuna sector, which lands half the national catch, these regulations could significantly improve sustainability in the Indo-Pacific region.

## Methodology

### Data collection

Under collaboration with a non-governmental organization (NGO) Masyarakat Dan Perikanan Indonesia (MDPI), SPOT Trace devices were voluntarily attached to 34 different hand line fishing vessels in three different provinces in Indonesia: West Nusa Tenggara (N. 15), East Nusa Tenggara (N. 11) and South of Celebes (N. 8). The target catch of these vessels are mainly tropical tunas: yellowfin tuna (*Thunnus albacares*), skipjack tuna (*Katsuwonus pelamis*), albacore (*Thunnus alalunga*), and big eye tuna (*Thunnus obesus*). The data were collected from August 2016 to January 2018 and include data from 70 fishing trips with a total 18,352 location messages (pings) recorded. Of these 70 fishing trips, 27 of them were included in a port sampling program to measure the catch composition, the length of main target species and also a total weight catch during the unloading. As part of the port sampling procedure by MDPI, interviews were carried out to record information including crew number, duration of the trip, and whether the AFADs were being used during the fishing trip. Each vessel was given a Vessel Identifier Code (VIC) based on the location of the fisher’s fishing base and the following was recorded: vessel size, trip duration, catch composition, and fishing gear/strategy.

The size of the boats in our study ranged between two and 29 Gross Tonnage (GT) while the duration of fishing trip ranged from several days to a maximum 1 month. The foraging strategy is mostly AFAD fishing where the fishers attach their vessels to the AFAD’s rope and use it as an anchor. Fishing operations then start directly from the anchored vessel or by deploying a small boat (e.g. canoe) to fish around the AFADs. Hence, a concentrated ping from the tracking data could indicate an AFAD. Another fishing strategy is to fish free swimming schools, away from FADs. This may involve fishers following dolphins and fishing along the animal’s trajectory. The GPS tracking devices on the vessels were set to transmit their position every hour. Due to the limited number of tracking devices available, devices were rotated across vessels, with different starting times and dates for each vessel, based on tracker availability.

### Spatial and statistical analysis

All data analysis was performed using the statistical language R version 4.0.3^36^. Each vessel trip was reconstructed based on departure and arrival from ports and matched to the port sampling data. The coordinate ping position and time generated from the tracking device were used to calculate the vessel speed, the bearing of the vessel and also the distance from the land. Later, the vessel position data from each fishing trip was then subsetted to positions that were at least two kilometres from land with a speed of less than one kilometre per hour. This slow speed indicated active fishing rather than transit as the hand line fishing vessel will attach their vessels to the AFAD and use it as an anchore to avoid drifting. However, stationary position can also indicate bait fishing as hand line fishing vessel also often catch small fish for later use as a bait to fish tuna. A key difference between these activities is that bait fishing does not occur at AFADs typically, and thus would not be expected to be in a consistent position across trips. Where bait fishing is done at an AFAD, for instance to replenish bait supplies during fishing for target species, it is equally viable as an indicator of the presence of an AFAD. For every subset of pings location, bathymetry data extracted from the GEBCO 30 arc second spatial resolution^49^ were assigned for later used in the cluster analyses.

Because AFADs have a main rope that is longer than the depth where they are anchored, AFADs have a radius of movement around the anchored position. With the assumption that the length of the main rope is twice the depth of the location^29^, the radius of AFAD movement can be obtained by using Pythagoras’ theorem (depth^2^ + surface radius^2^ = rope length^2^).

Spatial cluster analysis was then performed for each single trip tracking data by using the density-based spatial clustering of applications with noise (DB SCAN) algorithm, as implemented in the dbscan package in the R statistical language^50^. To apply the DBSCAN analysis, the maximum depth on each trip was used as a reference depth to measure the radius of cluster input. Each cluster output which has a minimum of 3 points then were classified as a suspected AFAD location and the mean of latitude and longitude were calculated to get a coordinate of suspected AFAD location. To determine the number of total suspected AFADs, each suspected AFAD coordinate from each trip was plotted visually. If there was more than one suspected AFADs from different fishing trip whose inter radius distance was located at a distance less than each point radius, it will be classified as a single suspected AFAD and be considered that the suspected AFAD was visited by more than one vessel or trips. A suspected AFAD position which had more than one trip visit, was then classified as a confirmed AFAD. A confirmed AFAD will also include any suspected AFAD from a fishing trip which stated “AFAD fishing” during port sampling interviews, even if the AFAD was not visited two or more times. These confirmed AFAD criteria enabled us to distinguish whether the stationary fishing was AFAD usage or bait fishing as both could happen in the same trip.

Apart from interviews during the port sampling, researcher ACW joined a fishing trip on board a hand line vessel in West Nusa Tenggara Province to check the accuracy of the AFAD detection approach. The trip last for 15 days with a fourteen GT vessel and seven crew members. SPOT Trace GPS units were carried during the fishing trip.

A 10 nautical mile circle was put on selected confirmed AFAD to provide information about AFAD density and to determine whether the deployment of AFADs by fishermen were abiding by current regulations. There is a minimum of ten nautical miles spacing required between AFADs by government decree. The distance of each AFAD from the nearest land was also calculated to understand AFAD position relative to the land.

Each AFAD detected using cluster analysis was then further evaluated to understand its use pattern. This was accomplished by evaluating the number of vessels visiting the same AFAD. The number of pings from each vessel in a particular AFAD was cumulatively added to analyse the length of tracking record of each AFADs. We estimated the minimum lifetime of the AFADs by assessing the elapsed time between the first and last visits to a given AFAD by any vessel. Since the deployment of vessel tracking devices are limited, AFADs could still exist but without many fishing vessels operating on the AFADs. We used the total elapsed time of the tracking device deployment relative to the elapsed time of use for vessels at an AFAD to determine if use had stopped due to removal of the trackers from the relevant vessels or whether the AFAD was potentially no longer in use. It is likely that these data substantially underestimate the average AFAD lifetime, due to the limited deployment length of tracking devices on vessels and the possibility of shifting use patterns. However, this tracking data provides a minimum AFAD lifetime estimate, which can be used to estimate the deployment rate required to maintain the standing stock of AFADs.

We also evaluated the relationship between the volume of fish landed and the characteristics of a fishing trip using generalized additive models with thin plate splines (GAMs) as implemented in the mgcv package in R^51^. Our hypothesis was that the more AFADs were visited during a trip, the less successful the fishing trip was. We assumed that if a vessel obtained enough catch from the first AFAD visited, the vessel would return to the fishing base (port) without fishing on other AFADs. We explored several model structures for this relationship. The models evaluated included smooth terms for the number of FAD visits and trip length alone, or in combination as additive terms, or as a two dimensional smooth. We included the vessel identity as a random intercept term, allowing average catch to vary by vessel. In the case of the two dimensional smooth, we were unable to include the random effect for vessel identity due to sample size constraints. We used the Akaike Information Criterion (AIC) to identify the best model from the models explored^52^. AIC provides an estimator of the relative quality of the model applied to a dataset. It estimates, in relative terms, the amount of information lost by a particular model (the higher quality model is that which loses less information). The lowest AIC value demonstrates the best-fit model. Note, however, that if values are within two points of one another, they are considered equally valid. To account for potential overfitting due to small sample sizes, a correction, AICc, is used.

## Conclusion

We found that voluntary vessel tracking data using GPS technologies, such as the SPOT Trace device, can provide high-quality information to support significant insight into fishing operations. This low-cost tool allowed identification of AFAD use based on vessel movement patterns, as well as providing a minimal estimate of the number and location of AFADs. Based on our data it appears that AFAD deployment and use patterns are generally not in compliance with existing regulations. We also found that we could extract useful information about catches from a simple analysis of AFAD use patterns and trip length. This is likely tied to the central place foraging style of the fishery, in which the use of large numbers of AFADs is an indicator of low catch rates. Clearly, there are also complex social relationships that affect AFAD use and sharing, which may well also translate into bias in port sampling, AFAD use patterns, and other metrics that are important for fisheries management.

## Supplementary Information

Supplementary Tables.
