# Supplementary material for: Detecting anchored fish aggregating devices (AFADs) and estimating use patterns from vessel tracking data in small-scale fisheries
Source: Sci Rep. 2021 Sep 9;11:17909. doi: 10.1038/s41598-021-97227-1 (PMC8429724; doi:10.1038/s41598-021-97227-1)
Supplement: Supplementary file 1 — Supplementary Tables. [file 41598_2021_97227_MOESM1_ESM.pdf]

Detecting Anchored Fish Aggregating Devices (AFADs) and Estimating Use Patterns from Vessel Tracking Data in Small-scale Fisheries

Ahmad Catur Widyatmoko[1,2,*], Britta Denise Hardesty[2,3,+], Chris Wilcox[2,3,+]

[1]CSIRO-UTAS Quantitative Marine Sciences PhD Program, Institute for Marine and Antarctic Studies, University of Tasmania, Hobart, Tasmania, Australia;
[2]Commonwealth Scientific and Industrial Research Organisation (CSIRO) Oceans and Atmosphere, Hobart, TAS 7000, Australia.
[3]Center for Marine Socioecology, University of Tasmania, Hobart, Tas 7000 Australia
[*]ahmadcatur.widyatmoko@utas.edu.au
[+]these authors contributed equally to this work

Supporting Information (SI)

Table. 1. Confirmed AFADs result from the DBSCAN analysis.

FAD ID	N. Cluster Pings	Depth (m)	Surface Radius (m)	First Date Visit Record	Last Date Visit Record	Distant to Land (km)	N. Vessel Visit	N. Trip Visit
1	160	-1402	2428.3	11/05/2017	17/05/2017	16.8	1	1
2	38	-2063	3573.2	19/04/2017	22/04/2017	41.9	1	1
3	1018	-1745	3022.4	18/09/2016	08/11/2016	38.7	3	5
4	430	-1243	2152.9	18/09/2016	30/10/2016	19.8	3	5
5	45	-1194	2068.0	26/09/2016	13/12/2016	21.9	2	3
6	149	-1550	2684.6	30/07/2017	06/09/2017	34.2	2	2
7	3	-1812	3138.4	20/07/2017	20/07/2017	27.1	1	1
8	93	-1347	2333.0	12/07/2017	17/07/2017	33.8	1	1
9	18	-1322	2289.7	13/07/2017	13/07/2017	30.3	1	1
10	63	-1559	2700.2	10/11/2016	18/07/2017	44.2	2	2
11	50	-1588	2750.4	20/07/2017	24/07/2017	21.2	1	1
12	21	-1519	2630.9	21/07/2017	22/07/2017	23.0	1	1
13	27	-1488	2577.2	24/10/2016	13/12/2016	23.0	1	2
14	7	-1453	2516.6	28/11/2016	28/11/2016	17.2	1	1
15	27	-700	1212.4	28/11/2016	14/12/2016	10.5	1	1
16	127	-1743	3018.9	30/11/2016	11/12/2016	37.6	1	1
17	112	-1534	2656.9	02/12/2016	12/12/2016	24.0	1	1
18	56	-2157	3736.0	03/08/2017	24/08/2017	60.5	2	3
19	98	-2555	4425.3	04/11/2016	17/09/2017	70.7	3	5
20	465	-2927	5069.7	13/10/2016	28/08/2017	91.4	3	4
21	1406	-1461	2530.5	15/09/2016	31/07/2017	55.4	3	10
22	41	-1343	2326.1	28/09/2016	29/12/2016	53.2	2	4
23	248	-1704	2951.4	27/10/2016	05/12/2016	53.3	2	3
24	39	-980	1697.4	09/11/2016	22/11/2016	14.1	1	1
25	44	-1909	3306.4	08/01/2017	20/07/2017	48.4	2	2
26	44	-1766	3058.8	31/07/2017	08/08/2017	50.1	2	2

27	18	-1385	2398.8	12/11/2016	16/11/2016	56.0	1	1
28	101	-1325	2294.9	19/12/2016	08/01/2017	61.5	1	2
29	126	-1960	3394.8	28/12/2016	07/08/2017	46.6	2	3
30	22	-1555	2693.3	29/12/2016	30/12/2016	56.1	1	1
31	19	-1115	1931.2	31/12/2016	31/12/2016	21.4	1	1
32	812	-1850	3204.2	10/08/2017	03/10/2017	28.1	2	2
33	242	-1798	3114.2	14/09/2016	06/06/2017	59.3	2	3
34	50	-1780	3083.0	31/08/2016	28/04/2017	60.7	2	2
35	39	-1959	3393.0	03/09/2016	09/05/2017	47.3	2	3
36	97	-1676	2902.9	04/09/2016	27/04/2017	51.5	2	2
37	174	-2919	5055.8	08/12/2016	18/12/2016	81.9	1	1
38	14	-2919	5055.8	16/12/2016	16/12/2016	89.7	1	1
39	3	-1752	3034.5	27/07/2017	01/08/2017	33.6	1	1
40	5	-2693	4664.4	04/08/2017	05/08/2017	84.5	1	1
41	519	-2384	4129.2	10/10/2016	16/11/2016	55.8	1	2
42	84	-1524	2639.6	21/04/2017	09/05/2017	33.9	1	2
43	8	-1015	1758.0	16/05/2017	16/05/2017	32.5	1	1
44	28	-1610	2788.6	21/05/2017	22/05/2017	66.5	1	1
45	259	-3179	5506.1	27/11/2016	09/05/2017	66.0	3	3
46	39	-1356	2348.6	01/09/2016	08/10/2016	19.2	2	2
47	41	-1448	2508.0	08/05/2017	13/05/2017	39.5	1	1
48	41	-1644	2847.4	08/05/2017	10/05/2017	47.0	1	1
49	20	-1764	3055.3	11/05/2017	12/05/2017	41.5	1	1
50	32	-1564	2708.9	08/10/2016	13/05/2017	39.4	2	2
51	64	-1553	2689.8	14/05/2017	15/07/2017	34.6	2	2
52	135	-1386	2400.6	20/05/2017	25/05/2017	54.5	1	1
53	3	-1371	2374.6	20/05/2017	20/05/2017	55.3	1	1
54	66	-662	1146.6	06/10/2016	29/10/2017	36.7	2	4
55	70	-679	1176.0	03/10/2016	10/11/2017	36.7	2	2
56	67	-1531	2651.7	09/10/2016	25/08/2017	31.9	3	4
57	20	-1396	2417.9	10/10/2016	27/07/2017	48.3	2	2
58	7	-1447	2506.2	28/04/2017	08/05/2017	69.0	1	1
59	205	-2972	5147.6	29/04/2017	20/07/2017	69.3	1	2
60	176	-2790	4832.4	30/04/2017	16/07/2017	65.2	1	2
61	16	-2652	4593.3	09/05/2017	09/05/2017	96.8	1	1
62	15	-1783	3088.2	19/05/2017	19/05/2017	38.6	1	1
63	166	-2382	4125.7	20/05/2017	27/05/2017	65.4	1	1
64	12	-2151	3725.6	27/05/2017	27/05/2017	100.2	1	1
65	12	-1905	3299.5	09/07/2017	09/07/2017	54.4	1	1
66	111	-1200	2078.4	02/11/2016	21/11/2016	22.0	1	2
67	112	-1062	1839.4	17/11/2016	18/11/2016	43.1	1	1
68	341	-1229	2128.6	15/07/2017	14/08/2017	22.3	2	4
69	195	-1558	2698.5	25/10/2017	31/10/2017	29.8	2	3

70	78	-1734	3003.3	19/08/2017	15/11/2017	39.5	2	2
71	393	-475	822.7	13/10/2017	02/11/2017	33.0	2	3
72	66	-632	1094.6	25/07/2017	18/08/2017	36.1	1	2

**VIC**	**Vessel Size (GT)**	**Trips ID**	**Trip Duration (Days)**	**N. Crew**	**N. FAD Visit Detected**	**Interview FAD use (Y/N)**	**Total catch (kg)**	**N. Individual Sample**		
								**SKJ***	**YFT***	**BET***
W049	5	W049_A	8	8	1	Y	842	NA	12	2
W051	6	W051_A	7	8	1	Y	179	NA	1	1
W053	5	W053_A	20	5	3	Y	1012	40	64	NA
	5	W053_B	21	3	2	Y	1125	14	96	NA
W061	5	W061_A	11	4	2	Y	1615	118	16	NA
	5	W061_B	21	4	1	Y	776	65	20	NA
W062	5	W062_A	7	7	2	Y	710	30	74	1
	5	W062_B	11	8	3	Y	649	NA	40	NA
W113	6	W113_A	15	5	6	Y	1224	108	4	NA
W115	5	W115_A	20	4	2	Y	1102	72	65	NA
	5	W115_B	18	4	6	Y	852	6	29	NA
W118	3	W118_A	11	9	3	Y	1658	NA	57	NA
	3	W118_B	12	9	1	Y	981	NA	45	NA
W122	5	W122_A	8	7	6	Y	393	8	96	NA
	5	W122_B	5	6	3	Y	1080	NA	1	NA
E012	5	E012_A	14	4	2	Y	1015	1	31	1
E045	6	E045_A	14	5	2	Y	2307	163	7	NA
E062	17	E062_A	9	3	2	Y	1582	101	96	8
	17	E062_B	10	4	1	Y	994	70	81	29
S008	2	S008_A	9	1	5	Y	125	NA	1	NA
	2	S008_B	7	1	2	Y	372	NA	11	NA
	5	S001_A	14	3	4	Y	189	NA	11	NA
SB001	5	SB001_A	13	3	3	Y	671	NA	8	NA
	5	SB001_B	15	3	3	Y	420	7	51	NA
SC002	3	SC002_A	7	2	3	Y	88	NA	3	NA
SC004	2	SC004_A	7	1	1	Y	137	NA	4	NA
SC007	3	SC007_A	7	1	2	Y	270	NA	38	NA

Tabel. 2. Port sampling data
**SKJ= Skipjack Tuna, YFT=Yellowfin Tuna, BET=Bigeye Tuna*
